# Association Between Age, Systemic Diseases and Oral Health Habits in Stomatological Teaching Hospital Patients: A Cross-Sectional Study

**DOI:** 10.3290/j.ohpd.c_2838

**Published:** 2026-10-21

**Authors:** Yongle Shi, Shufang Du, Chenxin Huang, Yaqin Li, Fan Liu

**Affiliations:** a Yongle Shi Researcher, Department of Medical Affairs, Outpatient Administration Office, State Key Laboratory of Oral Diseases, National Clinical Research Center for Oral Diseases, West China Hospital of Stomatology, Sichuan University, Chengdu, China. Direct participation (data collection, data analysis/interpretation), manuscript drafting (drafting the article, critical revision of the article for intellectual content).; b Shufang Du Researcher, Orthodontics, State Key Laboratory of Oral Diseases, National Clinical Research Center for Oral Diseases, West China Hospital of Stomatology, Sichuan University, Chengdu, China. Direct participation (data collection, data analysis/interpretation), manuscript drafting (drafting the article, critical revision of the article for intellectual content).; c Chenxin Huang Researcher, Southwestern University of Finance and Economics, Chengdu, Sichuan, China. Direct participation (data collection, data analysis/interpretation).; d Yaqin Li Researcher, School of Nursing, The Hong Kong Polytechnic University, Hong Kong, China. Work support (statistical analysis, funding acquisition, technical or material support, supervision, supportive contributions).; e Fan Liu Researcher, Department of Nursing, State Key Laboratory of Oral Diseases, National Clinical Research Center for Oral Diseases, West China Hospital of Stomatology, Sichuan University, Chengdu, China. Direct participation (data collection, data analysis/interpretation), work support (statistical analysis, funding acquisition, technical or material support, supervision, supportive contributions).

**Keywords:** elderly, oral health habits, risk management, stomatological hospitals, systemic diseases

## Abstract

**Objective:**

This study examined age-related disease patterns and health behaviours in dental outpatients to enhance risk assessment and guide clinical decision-making, providing strategies for managing medical risks in dental clinics.

**Methods and Materials:**

This cross-sectional study enrolled 2,241 consecutive patients from a tertiary stomatological teaching hospital between November 2024 and April 2025. Based on the Pre-Treatment Risk Assessment Questionnaire for Dental Outpatients, a validated questionnaire was used to assess general demographic characteristics (10 items), systemic disease and medication use history (36 items) and oral hygiene habits (9 items). Data were analysed using SPSS 23.0 and Python 3.9.

**Results:**

It showed that 12.5% of patients were aged ≥ 60 years. Elderly dental patients had a significantly higher prevalence of both single-system and multi-system diseases compared to other age groups (*p* < 0.001). Specifically, the population showed significantly greater proportions of sensory disorders, cardiovascular diseases, respiratory system diseases, urogenital disorders, gastrointestinal disorders, neurological disorders, and endocrine system diseases, medication use, and history of organ transplantation than younger patients, especially coronary heart disease, hypertension, cardiac arrhythmia, diabetes mellitus, thyroid dysfunction and osteoporosis than younger age groups (all *p* < 0.01). Furthermore, compared to younger patients, elderly patients had lower frequencies of toothbrushing, dental floss use, water flosser use, professional cleaning, and dental check-ups but exhibited reduced gingival bleeding and oral malodor(all *p* < 0.001).

**Conclusions:**

Elderly patients exhibit higher systemic comorbidities but poorer oral hygiene than younger individuals, necessitating integrated care with risk assessment and oral health education. These findings highlight the importance of incorporating geriatric medicine training to improve interdisciplinary collaboration.

With the growing public demand for oral healthcare, dental institutions are experiencing rapid development. The annual number of dental outpatient visits continues to rise, accompanied by evolving patient demographics characterised by increasing proportions of elderly and paediatric patients.^4,12
^ This demographic shift has elevated medical risks in dental outpatient settings.

Although life-threatening events in dental practice are rare, they remain a clinical reality.^20,24
^ Dental treatments frequently cause temporary impairment of essential functions including mastication, speech, and swallowing. These physiological challenges, combined with psychological stress, potential drug interactions, therapeutic interventions, and the patient’s underlying health status, may precipitate medical emergencies across all age groups during dental procedures.^20^ Studies estimate that two adverse events occur per 10,000 patients receiving local anaesthesia, and one cardiac arrest may arise per 638,960 dental patients.^17,24
^ Additionally, approximately 1 in 20 practitioners will perform cardiopulmonary resuscitation during their career.^7^


Oral health serves as both a critical component of systemic health and a determinant of ageing, engaging in bidirectional interactions with systemic diseases. This interdependence is particularly evident in vulnerable populations. Population ageing is a global phenomenon: virtually every country in the world is experiencing growth in the size and proportion of older persons in their population.^18,34
^ There were 703 million persons aged 65 years or over in the world in 2019, and the number of older persons is projected to double to 1.5 billion in 2050.^18^ The growing population of individuals with multiple chronic conditions, particularly elderly patients who typically present with systemic comorbidities and demonstrate poorer physical function and stress tolerance compared to younger individuals, is expected to increase significantly.^15,26
^ Paediatric patients frequently exhibit dental anxiety, often resulting in poor treatment compliance and elevated procedural risks.^31^ Studies indicate that 35% of systemic or comorbid conditions are associated with emergent risks during dental procedures, including hypoglycaemia, syncope, hypertensive crises, acute myocardial infarction, cardiac arrest, and anaphylactic shock.^2,6,10
^ Furthermore, patients with poorly controlled, unmonitored, or overlooked systemic conditions demonstrate increased susceptibility to medical emergencies during dental treatment.^9^ These treatment risks significantly impact patient care outcomes, lead to inefficient utilisation of dental resources, and pose serious threats to patient safety, all contributing to increased incidence of dental malpractice disputes. The Notice on Further Strengthening Medical Safety Management and Risk Prevention issued by the Chinese National Health Commission emphasises the critical need to enhance medical safety protocols and risk prevention measures to safeguard public health.^21^ Therefore, this study systematically analysed age-related patterns of systemic diseases and oral health behaviours among dental outpatients, aiming to provide clinically actionable insights for developing targeted risk mitigation strategies and refining risk assessment protocols for medically complex patients in contemporary dental practice, inform curriculum development in geriatric dentistry and optimise interdisciplinary collaboration, and ultimately enhance both patient care quality and clinical education outcomes.

## Methods and Materials

### Study Design

This cross-sectional study used a convenience sampling method at the largest oral healthcare education and clinical centre in southwestern China, a tertiary stomatological teaching hospital in Sichuan. As a specialised institution integrating medical services, education and research, the hospital is the primary training site for oral healthcare professionals, including undergraduates, postgraduates and resident trainees, while delivering comprehensive clinical services through its outpatient and emergency departments. The study encompassed multiple dental specialities, including oral emergency, prosthodontics, implantology, endodontics, oral surgery, orthodontics, periodontics, oral mucosal diseases, and geriatric dentistry. The Institutional Ethics Committee of our hospital approved this project, and the number was WCHSIRB-CT-2024-456.

### Participants

Participants were recruited from patients attending the outpatient and emergency departments of a tertiary dental hospital in Sichuan Province, China, from November 2024 to April 2025. The inclusion criteria comprised: (1) outpatient or emergency patients or their caregivers; (2) caregivers of paediatric patients (≤ 16 years); (3) caregivers of cognitively impaired patients; (4) patients or caregivers with intact cognitive and verbal abilities; (5) patients or caregivers who had given informed consent and voluntarily participated in this survey. Exclusion criteria were: (1) cases not requiring dental treatment, and (2) participants who withdrew from the study prematurely.

The minimum required sample size was determined by multiplying the 55 questionnaire items by 20 (n = 1,100), following established methodology for survey research. Accounting for a 20% anticipated invalid response rate, this was adjusted upward to 1,375 participants. The final study cohort comprised 2,241 participants, significantly exceeding both the minimum requirement and the adjusted target, thereby providing enhanced statistical power for the analysis.

### Assessment Questionnaire

The study used the finalised version of the Pre-Treatment Risk Assessment Questionnaire for Dental Outpatients, developed by the research team, as the basis for the survey instrument.^25^ It demonstrated excellent results in expert consultation: the effective recall rate reached 100%, with 100% expert participation. The authority coefficient was 0.83, and Kendall’s concordance coefficients ranged from 0.808 to 0.839 (*p* < 0.001), indicating statistically significant agreement among experts and confirming satisfactory content validity of the questionnaire.^25^ The questionnaire of this study comprised three dimensions: general demographic characteristics of patients, systemic disease history and medication use, and oral hygiene habits, totalling 55 items. Specifically, it included 10 items on demographic characteristics, 36 items on systemic disease history and medication use, and 9 items on oral hygiene habits. Preliminary assessment results showed good internal consistency, with overall and dimension-specific Cronbach’s alpha coefficients exceeding 0.80, indicating that the questionnaire was reliable.

#### Primary outcome

The status of systemic disease prevalence across different age groups in dental outpatients, measured by the number and type of systemic conditions reported in the pre-treatment risk assessment questionnaire.

#### Secondary outcomes

(1) The comparison of oral hygiene behaviours (toothbrushing frequency, dental floss use, water flosser use, professional cleaning, dental check-ups) across age groups; (2) The variable correlation between age, systemic disease burden, and oral health maintenance behaviours; (3) The identification of high-prevalence systemic diseases (coronary heart disease, hypertension, cardiac arrhythmia, diabetes mellitus, thyroid dysfunction, osteoporosis) specific to elderly dental patients.

### Quality Control Measures

Rigorous quality control measures were implemented across all study phases to ensure data reliability and validity. Participant selection strictly followed inclusion/exclusion criteria to maintain sample homogeneity while ensuring adequate representation across age, gender, and disease types. A pilot study (n = 50) informed questionnaire refinements to enhance validity and reliability. Standardised data collection protocols included comprehensive staff training, duplicate data entry, and meticulous verification procedures. Analytical rigour was maintained through appropriate statistical methods and thorough results verification.

### Statistical Analysis

Statistical analyses were conducted using IBM SPSS Statistics (version 23.0) and Python (version 3.9). Categorical variables were expressed as frequencies and percentages (n, %). Inter-group comparisons were conducted using Pearson’s chi-square tests for contingency tables where all expected cell frequencies were ≥ 5. When more than 20% of cells had expected frequencies < 5, Fisher’s exact test (derived from the hypergeometric distribution) was employed to maintain statistical validity in assessing associations between categorical variables.

## Results

### General Demographic Characteristics

The study found that the majority of dental patients were between 19 and 59 years old (76.8%), with a higher proportion of females (64.9%). Most participants were urban residents (87.1%), and over half held at least a bachelor’s degree (54.6%). Nearly all patients (99.3%) were ambulatory upon arrival at the clinic, while 59.4% attended without a caregiver. Urban employee health insurance was the most prevalent coverage type (62.1%). Regarding systemic diseases, 13.6% of patients presented with one systemic disease, and 12.0% had two or more (Table 1).

**Table 1 Table1:** General demographic characteristics of participants (n = 2241)

Variables	Classification	Numbers	Percent (%)
Departments	Dental Emergency Department	28	1.2
	Prosthodontics	254	11.3
	Department of Oral Implantology	27	1.2
	Orthodontics	487	21.7
	Oral Surgery	531	23.7
	Operative Dentistry and Endodontics	341	15.2
	Periodontics	85	3.8
	Department of Geriatric Dentistry	272	12.1
	Department of Oral Medicine	104	4.6
	Preventive Dentistry	73	3.3
	General Dentistry	39	1.7
Nationality	China	2237	99.8
	Others	4	0.2
Ethnicity	Han Chinese	2159	96.3
	Others	82	3.7
Residence	Urban	1951	87.1
	Rural	290	12.9
Age (years old)	0–18	238	10.6
	19–59	1722	76.8
	≥ 60	281	12.5
Gender	Male	787	35.1
	Female	1454	64.9
Education level	Primary education or lower	183	8.2
	Junior high school	216	9.6
	Senior high school	234	10.4
	College diploma	384	17.1
	Bachelor’s degree or above	1224	54.6
Admission method	Walk-in	2225	99.3
	Assisted admission	16	3.7
Caregiver status	Present	909	40.6
	Absent	1332	59.4
Medical insurance	Urban Employee Medical Insurance	1391	62.1
	Rural Cooperative Medical Insurance	171	7.6
	Others	679	30.3
Systemic diseases	No	1668	74.4
	1	305	13.6
	2 or more	268	12.0


### Comparison of Systemic Diseases Across Age Groups

The results demonstrated that elderly dental patients had a significantly higher prevalence of both single-system and multi-system diseases compared with other age groups (χ^2^ = 242.751, *p* < 0.001; Table 2). Specifically, this population showed significantly greater proportions of sensory disorders, cardiovascular diseases, respiratory system diseases, urogenital disorders, gastrointestinal disorders, neurological disorders, and endocrine system diseases than younger patients (all *p* < 0.001; Fig 1). Furthermore, the elderly group exhibited higher rates of both chronic medication use and history of organ transplantation (*p* < 0.001; Fig 1).

**Table 2 Table2:** Comparison of the number of systemic diseases across age groups (n = 2241)

Variables	Classification	Age stratification (years) [n(%)]			X^2^	*p*
		0–18 (n1 = 238)	19–59 (n2 = 1722)	≥ 60 (n3 = 281)		
Systemic diseases	No	212 (89.1)	1340 (77.8)	116 (41.3)	242.751	< 0.001
	1	14 (5.9)	229 (13.3)	62 (22.1)		
	2 or more	12 (5.0)	153 (8.9)	103 (36.7)		


**Fig 1 Fig1:**
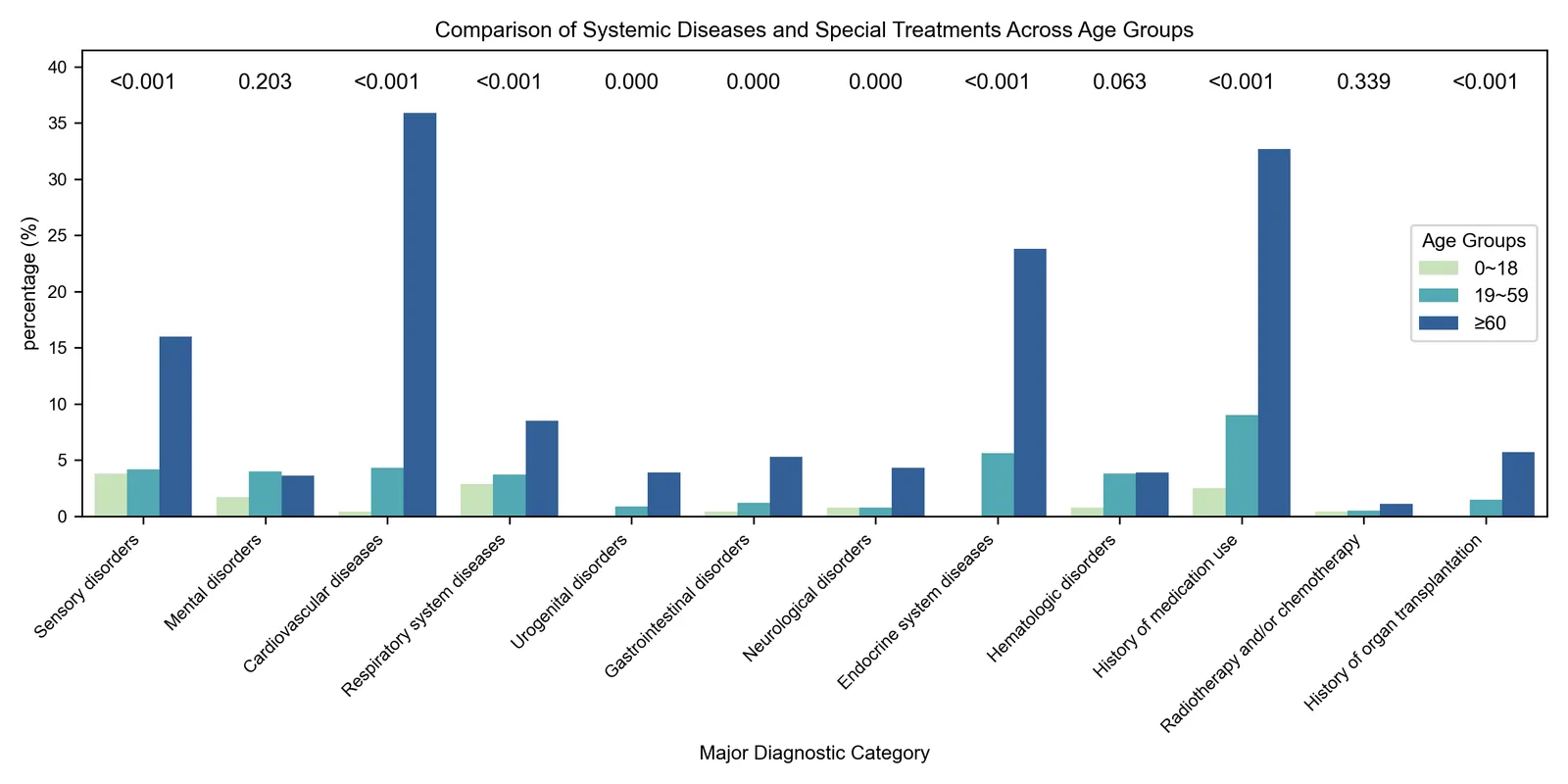
Comparison of systemic diseases and special treatments across age groups.

### Comparison of High-Prevalence Diseases Across Age and Department Groups

The findings showed that elderly dental patients had a much higher rate of systemic diseases, including coronary heart disease, hypertension, cardiac arrhythmia, diabetes mellitus, thyroid dysfunction and osteoporosis, than younger age groups (all *p* < 0.01; Fig 2). Further analysis revealed no correlation between high-prevalence diseases in the patient group and the departments they visited (Fig 3).

### Comparison of Oral Health Behaviours Across Age Groups

Significant age-related differences (all *p* < 0.001) were observed in oral health behaviours, including toothbrushing, flossing, water flosser use, professional cleaning, dental visits, gingival bleeding, and oral malodor. Compared to younger patients, elderly patients had lower frequencies of toothbrushing, dental floss use, water flosser use, professional cleaning, and dental check-ups but exhibited reduced gingival bleeding and oral malodor (Fig 4).

## Discussions

The study revealed that elderly patients in dental outpatient and emergency settings exhibited significantly higher prevalence rates of systemic diseases such as hypertension, coronary heart disease, arrhythmia, diabetes, and osteoporosis compared to other age groups, which aligned with existing literature.^3^ This phenomenon was attributed to age-related physiological decline, where the progressive deterioration of immune function and physiological systems in the elderly population substantially elevated the risk of multiple chronic diseases.^27^


Oral health served as both a critical component of systemic health and a determinant of ageing, engaging in bidirectional interactions with systemic diseases.^33^ The oral microbiome, a diverse and complex ecosystem comprising bacteria, fungi, and viruses, played a pivotal role in maintaining oral-systemic health. Its composition and dynamics were intricately linked to physiological processes.^8,28
^ Disruption of this delicate balance, leading to oral dysbiosis, could precipitate oral diseases including caries, gingivitis, periodontitis, halitosis, and oral candidiasis while exerting profound systemic effects on gastrointestinal, cardiovascular, endocrine, and neurological systems.^5,11
^ Emerging evidence associated oral health with Alzheimer’s disease, cancer, inflammatory bowel disease, osteoporosis, pneumonia, and rheumatoid arthritis.^24,32
^ Salivary gland disorders correlated with systemic conditions such as hypertension, diabetes, and HIV, while oral infections including periodontitis, gingivitis, and caries influenced the pathogenesis of cardiovascular and cerebrovascular diseases.^23,32
^ Alzheimer’s disease, a neurodegenerative disorder predominantly affecting individuals over 65, demonstrated strong ties to oral microbiota composition.^14^ In oncology, specific bacterial strains were implicated in cancer progression,^23^ and inflammatory bowel disease exhibited links to oral dysbiosis.^22^ Diabetes, characterised by hyperglycemia and inflammation, fostered microbial imbalance, with observed oral microbiota alterations potentially contributing to diabetes-related complications.^30^ Notably, periodontitis extended beyond localised risks, emerging as a systemic hazard for cardiovascular disease, diabetes, and cancer.^13,23
^ The oral microbiome and cardiovascular health shared a bidirectional relationship mediated by systemic inflammation and immune activation.^23^ Thus, cardiovascular conditions including hypertension and arrhythmia, and endocrine disorders such as diabetes and thyroid dysfunction, which were closely tied to oral health, interacted dynamically with primary oral conditions. Systemic comorbidities were prevalent among elderly dental patients, with acute manifestations including shock and cardiovascular emergencies posing life-threatening risks during treatment. Studies highlighted elevated pretreatment risks of acute myocardial infarction or cardiac arrest in this population.17 These findings necessitated a paradigm shift in recognising oral-systemic health interdependencies. Comprehensive pretreatment assessment of systemic disease history was imperative to mitigate emergent risks throughout the dental care continuum.

The study identified limitations in oral hygiene behaviours among elderly patients in dental outpatient and emergency departments. Compared to younger patient groups, the elderly exhibited lower frequencies of toothbrushing, dental floss use, and professional dental cleanings. Poor oral hygiene habits played a pivotal role in shaping the oral microbial ecosystem. Insufficient toothbrushing and flossing promoted plaque accumulation, creating a favourable environment for pathogenic bacterial proliferation and leading to oral microbiome dysbiosis.^1^ The inevitable process of physiological ageing presented multiple challenges to maintaining oral hygiene in the elderly. Ageing was often accompanied by multiple chronic comorbidities that not only diminished physical function but also interfered with proper implementation of oral hygiene measures. Sensory impairments, including declining vision, reduced tactile sensitivity, and diminished dexterity, made routine oral cleaning practices such as brushing and flossing significantly more difficult for the elderly compared to younger individuals.^16^ Psychological and social factors contributed to notable differences in oral health perception and behaviours across age groups. Elderly patients, particularly those with declining physical function, cognitive impairment, and limited oral health awareness, faced restricted access to reliable and effective oral health information due to factors including psychological resilience, attitudes toward oral health, personal values, and socioeconomic circumstances.^19^ This knowledge gap directly resulted in inadequate oral hygiene practices, increasing their susceptibility to oral diseases. Additionally, elderly patients frequently encountered difficulties due to oral discomfort or physical limitations. Restricted mobility hampered their ability to clean hard-to-reach areas effectively, while tooth loss not only impaired chewing function but also compromised oral structural integrity, making thorough oral hygiene maintenance even more challenging. These factors acted as catalysts, further exacerbating the prevalence of oral health issues. Therefore, enhancing oral health awareness and behaviours among elderly patients through personalised oral health education was crucial for improving their oral hygiene practices and overall oral health. Importantly, the parallel finding of inadequate oral hygiene behaviours suggests these medical risk assessments should be complemented by targeted educational interventions specifically designed for elderly patients’ unique needs and limitations. Furthermore, the complex bidirectional relationship between oral and systemic health in ageing populations demands coordinated management through structured collaboration between dental and general medical professionals.^29,35
^ Only through such interdisciplinary integration can we adequately address both the medical risks and oral health challenges facing this vulnerable patient population. From a teaching perspective, the high prevalence of systemic conditions in elderly dental patients also highlights the urgent need to incorporate geriatric medicine training into dental curricula and to develop standardised patient simulations for the management of oral-systemic diseases in clinical assessments.

## Conclusions

This study reveals a dual challenge in geriatric dental care: elderly patients in tertiary hospital settings demonstrate both higher systemic comorbidity burdens and poorer oral hygiene maintenance compared to younger populations. These interconnected findings necessitate integrated clinical approaches combining comprehensive medical risk assessment with tailored oral health education, all supported by strengthened collaboration between dental and medical professionals to properly manage the oral-systemic health interplay in ageing patients. However, three limitations should be acknowledged when interpreting these findings. First, the single-centre design and convenience sampling method may limit generalisability and introduce selection bias, as the cohort represents patients with treatment needs who were willing and able to complete the questionnaire; patients without treatment needs were excluded, which may affect the representativeness of the overall dental-seeking population. Second, the cross-sectional design precludes causal inference regarding the relationship between age, systemic disease burden, and oral health behaviours. Third, reliance on self-reported data for both systemic diseases and oral health behaviours, without clinical oral disease metrics such as periodontal indices (CPI, BOP), caries experience (DMFT), or mucosal pathology screening, precludes direct correlation between systemic comorbidities and specific oral pathologies. Future prospective multicenter investigations, incorporating integrated clinical-questionnaire designs and simultaneously collecting periodontal indices, caries data, and mucosal screening results across diverse treatment-need populations, are warranted to validate these findings, establish comprehensive oral-systemic correlations, and refine evidence-based management strategies for this vulnerable population.

### Acknowledgements

#### Funding

This study was supported by (1) the Soft Science Research Fund of West China School of Stomatology, Sichuan University (No. RD-01-201906), and (2) the Popular Science Project of the Sichuan Provincial Department of Science and Technology (grant number 2024JDKP0106).

### Ethics Approval Statement

The Institutional Ethics Committee of West China Hospital of Stomatology, Sichuan University approved this project, and the number was WCHSIRB-CT-2024-456.

### Declaration of Competing Interest

The authors declare no competing interests.

#### Data sharing statement

All the data and information in this study are available from the corresponding author upon reasonable request.

## References






